# Komplikationen der akuten Otitis media

**DOI:** 10.1007/s00106-026-01788-4

**Published:** 2026-06-30

**Authors:** Lukas Schmutzler, Matthias Santer, Daniel Dejaco, Benedikt Hofauer, Joachim Schmutzhard

**Affiliations:** Universitätsklinik für Hals‑, Nasen- und Ohrenheilkunde, Medizinische Universität Innsbruck, Anichstraße 35, 6020 Innsbruck, Österreich

**Keywords:** Mittelohr, Fazialisparese, Sinusvenenthrombose, Mastoiditis, Abszess, Middle ear, Facial paralysis, Cerebral venous sinus thrombosis, Mastoiditis, Abscess

## Abstract

Komplikationen der akuten Otitis media (AOM) sind selten, können jedoch intratemporal oder intrakraniell auftreten. Klinisch relevante Mastoiditiden zeigen sich meist durch subperiostale Abszesse, während subklinische Verschattungen der Mastoidzellen häufig auftreten, aber keine spezifische Therapie erfordern. Weitere mögliche Komplikationen umfassen Labyrinthitis, Fazialisparese, intratemporale Abszesse, Sinusvenenthrombosen oder Meningitiden. Die Diagnostik basiert auf klinischer Untersuchung, Laborparametern und gezielter Bildgebung. Intratemporale Komplikationen werden primär antibiotisch behandelt. Chirurgische Eingriffe sind bei Abszessbildung oder Therapieversagen erforderlich. Intrakranielle Komplikationen bedürfen meist einer interdisziplinären operativen Versorgung. Die vorliegende Übersicht bietet praxisnahe Empfehlungen zu Differenzierung, Diagnostik und Therapie und soll die Aufmerksamkeit für potenziell schwerwiegende Verläufe schärfen.

## Abstract (en)

Complications of acute otitis media (AOM) are rare but can occur intratemporally or intracranially. Clinically relevant mastoiditis usually presents with subperiosteal abscesses, whereas subclinical opacification of the mastoid cells is common but does not require specific treatment. Other potential complications include labyrinthitis, facial nerve palsy, intratemporal abscess, cerebral venous sinus thrombosis, or meningitis. Diagnosis relies on clinical examination, laboratory tests, and targeted imaging. Intratemporal complications are primarily treated with antibiotics. Surgical intervention is indicated in cases of abscess formation or treatment failure. Intracranial complications often necessitate interdisciplinary surgical management. This review provides practical guidance on differentiation, diagnosis, and treatment, highlighting the importance of recognizing potentially severe courses.

## Lernziele

Nach Lektüre dieses Beitrags…

- sind Sie in der Lage, die wichtigsten Komplikationen der akuten Otitis media zu erkennen und zu unterscheiden,
- verstehen Sie die Risikofaktoren und pathophysiologischen Mechanismen der Komplikationen,
- können Sie klinische Warnzeichen identifizieren und eine gezielte Diagnostik einleiten,
- wissen Sie, Therapie- und Managementoptionen praxisnah anzuwenden und interdisziplinär abzustimmen.

## Kasuistik

Ein 4‑jähriges Mädchen wurde mit persistierendem Fieber bis 39,5 °C seit 5 Tagen, akuten Ohrenschmerzen und **retroaurikulärer Rötung**

Retroaurikuläre Rötung

vorgestellt. Die Familie berichtete über **rezidivierende Mittelohrentzündungen**

Rezidivierende Mittelohrentzündungen

ohne bisherige schwerwiegende Komplikationen. Klinisch zeigte sich eine Schwellung hinter dem rechten Ohr, das Trommelfell war stark gerötet und vorgewölbt. Zudem bestand eine **Fazialisparese rechts**

Fazialisparese rechts

(House-Brackmann Grad II). Laboruntersuchungen zeigten erhöhte Entzündungsmarker. Eine Computertomographie (CT) des Felsenbeins bestätigte eine Mastoiditis mit **subperiostalem Abszess**

Subperiostaler Abszess,

jedoch ohne intrakranielle Ausbreitung. Initial wurde eine i.v.-Breitbandantibiotikatherapie eingeleitet und eine Paukendrainage zur Druckentlastung und Entfernung von Mittelohrerguss durchgeführt. Bei persistierender Symptomatik und **fehlender Besserung**

Fehlende Besserung

der Fazialisparese erfolgte schließlich eine **Mastoidektomie**

Mastoidektomie.

Diese diente der Drainage der entzündeten Mastoidzellen und der indirekten Entlastung des N. facialis durch Entfernung des entzündeten Gewebes. Unter enger Verlaufskontrolle besserten sich die Symptome rasch, die Fazialisparese bildete sich zurück, und die Entzündungswerte normalisierten sich.

## Epidemiologie und Risikofaktoren

Die Otitis media umfasst ein Spektrum von Mittelohrentzündungen: die **akute Otitis media**

akute Otitis media

(AOM) mit plötzlichem Einsetzen von Ohrenschmerzen, Fieber und Trommelfellvorwölbung, die **Otitis media mit Erguss**

Otitis media mit Erguss

(OME) ohne akute Entzündung sowie chronische Formen wie die **chronisch suppurative Otitis media**

Chronisch suppurative Otitis media

(CSOM) mit persistierender Trommelfellperforation und Otorrhö oder das **Cholesteatom**

Cholesteatom

als keratinisierende chronische Entzündung des Epitympanums. Während die AOM die entscheidende Entität für akute suppurative Komplikationen darstellt, unterscheiden sich OME und chronische Otitiden in Risikoprofil und Verlauf [1, 2, 3].

Die AOM ist eine der häufigsten Infektionen im Kindesalter, mit einem Höhepunkt zwischen dem 6. Lebensmonat und dem 2. Lebensjahr. Etwa 80 % der Kinder erleiden bis zum dritten Lebensjahr mindestens eine Episode. Bis zum siebten Lebensjahr haben ungefähr 40 % der Kinder 6 oder mehr Episoden durchgemacht. Bei Erwachsenen ist die AOM seltener als im Kindesalter, bleibt aber insbesondere bei Patienten mit Begleiterkrankungen klinisch relevant. Prädisponierend wirken **Tubenventilationsstörungen**

Tubenventilationsstörungen

bei Erwachsenen sowie **adenoide Vegetationen**

Adenoide Vegetationen

im Kindesalter [1, 2, 3, 4].

Komplikationen der AOM sind insgesamt selten. In einer großen US-amerikanischen Notaufnahmedatenbank trat nur bei etwa 0,26 % der AOM-Fälle eine Komplikation auf, wobei Mastoiditis, Labyrinthitis und Fazialisparese die am häufigsten dokumentierten intratemporalen Komplikationen waren [5]. Intratemporale Komplikationen überwiegen gegenüber intrakraniellen Komplikationen, wie Meningitis, Sinusvenenthrombosen oder Abszessbildungen. Bei Kindern ist insbesondere *Streptococcus pneumoniae* der häufigste Erreger bei suppurativen Komplikationen. **Risikofaktoren**

Risikofaktoren

für komplizierte Verläufe umfassen ein jüngeres Alter, ein unreifes oder **geschwächtes Immunsystem**

Geschwächtes Immunsystem,

persistierende Symptome, rezidivierende AOM, Infektionen mit virulenten oder antibiotikaresistenten Erregern, unzureichend behandelte Episoden sowie **Impflücken**

Impflücken,

insbesondere gegen Pneumokokken. Trotz dieser Risikofaktoren bleibt die Inzidenz von Komplikationen gering, wie in populationsbasierten Studien gezeigt wurde [6, 7, 8, 9, 10].

### Merke

Komplikationen der AOM sind insgesamt selten, treten aber v. a. bei kleinen Kindern und Patienten mit Risikofaktoren auf.

## Pathophysiologie

Komplikationen der AOM entstehen durch die Ausbreitung der Infektion auf benachbarte Strukturen und durch individuelle anatomische oder immunologische Faktoren des Patienten. Intratemporale Komplikationen treten auf, wenn die Entzündung vom Mittelohr auf Mastoidzellen, Innenohr oder den Verlauf des N. facialis übergreift. Dabei kann es infolge einer Ostitis der mastoidalen Kortikalis zur **Knochenarrosion**

Knochenarrosion

und schließlich zur Ausbildung eines subperiostalen Abszesses kommen. Zudem kann es zum Eindringen bakterieller oder entzündlicher Mediatoren in das Innenohr oder zu einer Schädigung des Fazialisnervs führen.

### Merke

Intratemporale Komplikationen entstehen durch direkte Ausbreitung der Infektion auf Mastoid, Innenohr oder N. facialis.

Die Pathophysiologie der Fazialisparese ist multifaktoriell: Diskutiert werden **direkte Infektionsausbreitung**

Direkte Infektionsausbreitung

über knöcherne Dehiszenzen, Osteitis des Falloppio-Kanals oder ein entzündlich bedingtes Ödem mit Ischämie des Nervs. Neurotoxische, immunvermittelte und **virale Mechanismen**

Virale Mechanismen

sind weitere mögliche Ursachen [11, 12]. Intrakranielle Komplikationen entstehen durch Infektionsausbreitung über venöse Gefäße oder **knöcherne Lücken**

Knöcherne Lücken

und können Abszesse, Sinusthrombosen oder Meningitiden verursachen. Sie sind meist mit erhöhter Morbidität verbunden und erfordern frühzeitige Erkennung sowie gezielte diagnostische und therapeutische Maßnahmen [13, 14, 15, 16].

### Merke

Intrakranielle Komplikationen erfolgen meist über venöse oder knöcherne Wege und können Abszesse, Sinusvenenthrombosen oder Meningitiden verursachen.

## Klinische Darstellung der wichtigsten Komplikationen

### Intratemporal

#### Mastoiditis

**Abb. 1:**
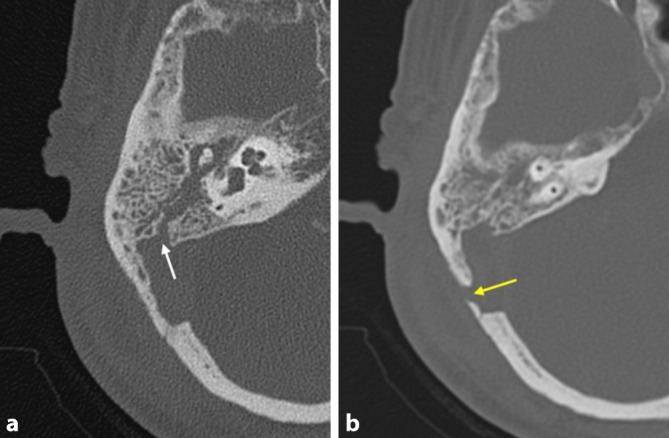
Axiale Computertomographie (CT). Rechtsseitige abszedierende Mastoiditis mit **a** dorsalem Kortikalisdefekt der Mastoidzellen (*weißer Pfeil*) und **b** knöchernem Defekt dorsal der Mastoidzellen (*gelber Pfeil*) mit Fortleitung der Entzündung nach extrakraniell

Die Mastoiditis bei der AOM (Abb. 1) entsteht durch die Ausbreitung der Entzündung vom Mittelohr auf die **pneumatisierten Mastoidzellen**

Pneumatisierte Mastoidzellen.

Eine mastoidale Beteiligung ist bei jeder AOM zu erwarten. Klinisch fällt v. a. die retroaurikuläre, **teigige Schwellung**

Teigige Schwellung

auf, die stets durch einen Subperiostalabszess verursacht wird. Typische Begleitzeichen sind **persistierendes Fieber**

Persistierendes Fieber,

zunehmende Ohrenschmerzen, Rötung, **Druckdolenz**

Druckdolenz

und das Abstehen der Ohrmuschel. Bei Kleinkindern können zusätzlich Reizbarkeit oder Trinkschwäche auftreten.

#### Komplizierte Mastoiditis und Abszesse

Bei sich ausbreitender Mastoiditis können **extratemporale Abszesse**

Extratemporale Abszesse

entstehen, deren Lokalisation von der Richtung der Infektionsausbreitung abhängt (z. B. subperiostaler Abszess bei lateraler Kortikalisperforation, Bezold-Abszess mit Ausbreitung in die tiefen Halsweichteile entlang des M. sternocleidomastoideus, Citelli-Abszess mit dorsookzipitaler Ausbreitung oder Muret-Abszess mit Ausbreitung in die Fossa digastrica). Selten kann die Infektion die Felsenbeinspitze betreffen, was zum **Gradenigo-Syndrom**

Gradenigo-Syndrom

führen kann, das durch die klassische Trias aus Otitis media/Mastoiditis, Trigeminusschmerz und Abduzensparese gekennzeichnet ist [17, 18].

#### Labyrinthitis

Die Labyrinthitis entsteht durch toxische oder bakterielle Ausbreitung aus dem Mittelohr ins **Innenohr**

Innenohr,

meist über das runde oder ovale Fenster im Rahmen einer entzündlich **gesteigerten Permeabilität**

Gesteigerte Permeabilität

der Membranen oder bei erosiven Veränderungen der knöchernen Begrenzungen. Klinisch dominieren **akuter Drehschwindel**

Akuter Drehschwindel,

Übelkeit, Erbrechen, Nystagmus und **sensorineurale Hörminderung**

Sensorineurale Hörminderung.

Die **seröse Form**

Seröse Form

ist meist reversibel und hat oft einen leichteren Verlauf, während die **eitrige Form**

Eitrige Form

ein hohes Risiko für **bleibende Hörschäden**

Bleibende Hörschäden

und persistierende Gleichgewichtsstörungen hat. In diesen Fällen sind eine rasche diagnostische Abklärung und therapeutische Eskalation erforderlich. Besonders bei Kindern kann die Symptomatik schwer erkennbar sein – hier sind auffällige Gangunsicherheit, **häufiges Hinfallen**

Häufiges Hinfallen

oder starke Irritation Hinweise [19, 20].

#### Fazialisparese

Mehrere Hypothesen führen die Entstehung einer **peripheren Fazialisparese**

Periphere Fazialisparese

auf ein Ödem, **erhöhte Druckverhältnisse**

Erhöhte Druckverhältnisse

im Fazialiskanal oder direkte **toxische Effekte**

Toxische Effekte

der Infektion zurück [11, 12]. Klinisch zeigt sich eine einseitige Schwäche der mimischen Muskulatur, eingeschränkter Lidschluss und **Mundwinkelabsinken**

Mundwinkelabsinken.

Der Schweregrad kann variieren – von leichter asymmetrischer Mundwinkelbewegung bis zu kompletter Lähmung der betroffenen Gesichtshälfte und wird klinisch anhand des **House-Brackmann-Scores**

House-Brackmann-Score

(Grad I–VI) beurteilt. Differenzialdiagnostisch ist insbesondere die Abgrenzung zu zentralen Fazialisparesen relevant. Periphere Fazialisparesen betreffen typischerweise die gesamte ipsilaterale Gesichtshälfte einschließlich Stirnmuskulatur und Lidschluss, können klinisch jedoch – insbesondere bei inkompletter Ausprägung (z. B. House-Brackmann Grad II–III) – auch nur als partielle Mundastschwäche imponieren. Zentrale Fazialisparesen zeigen dagegen meist eine Aussparung der Stirnmotorik [19, 21].

##### Merke

Periphere Fazialisparesen betreffen gewöhnlich auch Stirnmuskulatur und Lidschluss. Bei zentralen Fazialisparesen wird meist die Stirnmotorik ausgespart.

### Intrakraniell

#### Meningitis

Otogene Meningitiden zählen zu den häufigsten intrakraniellen Komplikationen der AOM und entstehen durch direkte oder **hämatogene Ausbreitung**

Hämatogene Ausbreitung

[22, 23]. Klinische Warnzeichen sind Fieber, starke Kopfschmerzen, **Nackensteife**

Nackensteife

(Meningismus) sowie Bewusstseinsveränderungen [23]. Insbesondere bei Säuglingen und Kleinkindern erfordert das variable klinische Bild eine sorgfältige Untersuchung und **frühzeitige Bildgebung**

Frühzeitige Bildgebung.

#### Intrakranielle Abszesse

**Abb. 2:**
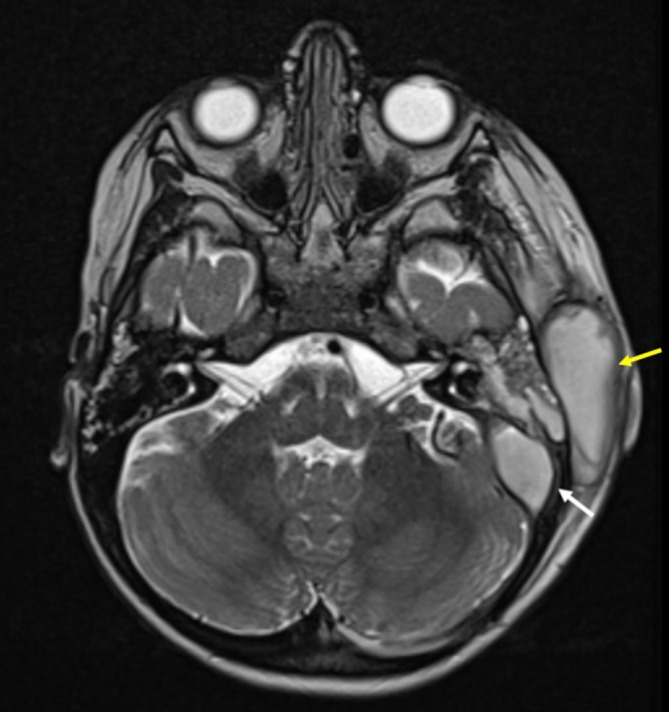
Axiale Magnetresonanztomographie (MRT; T2-gewichtet). Abszedierende Mastoiditis mit Durchbruch nach intrakraniell und Ausbildung eines epiduralen Abszesses (*weißer Pfeil*); außerdem extrakranielle (subperiostale) Abszessausdehnung (*gelber Pfeil*)

Intrakranielle Abszesse können epidural, subdural oder intrazerebral auftreten (Abb. 2). Typische Symptome umfassen Kopfschmerzen, fokalneurologische Defizite, **Vigilanzminderung**

Vigilanzminderung

und **epileptische Anfälle**

Epileptische Anfälle.

Bei jüngeren Kindern können anfänglich unspezifische Symptome wie Reizbarkeit oder Appetitlosigkeit auftreten. Intrakranielle Abszesse zählen neben der Meningitis zu den relativ häufigen intrakraniellen Komplikationen und erfordern bei Verdacht eine frühzeitige Bildgebung [22, 23, 24].

#### Sinusvenenthrombose

Die laterale Sinusvenenthrombose zählt zu den seltenen, aber relevanten intrakraniellen Komplikationen der AOM. Klinische Warnzeichen können anhaltende Kopfschmerzen, Fieber, Papillenödem und **Hirnnervenfunktionsstörungen**

Hirnnervenfunktionsstörungen,

insbesondere des **N. abducens**

N. abducens,

sein.

##### Merke

Intratemporale und intrakranielle Komplikationen sind selten, können aber schwer verlaufen.

Bei Kindern kann die Symptomatik unspezifisch oder maskiert auftreten, sodass eine sorgfältige klinische Beurteilung und frühzeitige Bildgebung entscheidend sind [7, 23, 24].

##### Merke

Warnzeichen wie anhaltende Kopfschmerzen, Fieber, retroaurikuläre Schwellung oder neurologische Auffälligkeiten erfordern sofortige Diagnostik und interdisziplinäres Management.

## Diagnostik

Die frühzeitige Erkennung von AOM-Komplikationen ist entscheidend, um Morbidität und Mortalität zu reduzieren. Die Diagnostik umfasst klinische Untersuchung, Labordiagnostik und gezielte Bildgebung. Neben klassischen AOM-Symptomen wie Otalgie, Fieber und Hörminderung sind insbesondere Warnzeichen für komplizierte Verläufe relevant: persistierendes oder **progredientes Fieber**

Progredientes Fieber

trotz Therapie, ausgeprägte retroaurikuläre Schwellung mit Abstehen der Ohrmuschel, neu aufgetretene Fazialisparese, Schwindel, **starke Kopfschmerzen**

Starke Kopfschmerzen,

Meningismus oder weitere neurologische Auffälligkeiten. **Neurologische Symptome**

Neurologische Symptome

gelten als klare Indikation zur **unverzüglichen Bildgebung**

Unverzügliche Bildgebung.

Laborparameter wie C‑reaktives Protein (CRP), Leukozytenzahl und Procalcitonin (PCT) spiegeln das Entzündungsausmaß wider, erlauben jedoch keine sichere Differenzierung zwischen unkomplizierten und komplizierten Verläufen. Bei Verdacht auf systemische Infektion oder **Sepsis**

Sepsis

sollten vor Beginn einer antibiotischen Therapie **Blutkulturen**

Blutkulturen

abgenommen werden.

### Merke

Die Kombination aus klinischer Untersuchung, Laborparametern und gezielter Bildgebung ermöglicht eine frühe Differenzierung intratemporaler und intrakranieller Komplikationen.

Die Wahl des bildgebenden Verfahrens richtet sich nach dem klinischen Verdacht. Bei intratemporalen Komplikationen wird primär die **kontrastmittelgestützte Computertomographie**

Kontrastmittelgestützte Computertomographie

(CT) des Felsenbeins empfohlen, da sie knöcherne Destruktionen, **Mastoidzellverschattungen**

Mastoidzellverschattungen

und subperiostale Abszesse zuverlässig darstellt. Bei Verdacht auf intrakranielle Komplikationen, wie Abszesse oder Sinusvenenthrombosen, gilt die **kontrastmittelgestützte Magnetresonanztomographie**

Kontrastmittelgestützte Magnetresonanztomographie

(MRT) als Methode der Wahl, da sie **Weichteilstrukturen**

Weichteilstrukturen,

meningeale Beteiligungen und venöse Sinus sensitiver abbildet als die CT. Bei Verdacht auf eine **Sinusvenenthrombose**

Sinusvenenthrombose

sollte ergänzend eine **MR-Venographie**

MR-Venographie

durchgeführt werden [6, 7, 21, 23, 24, 25, 26, 27]. Im Kindesalter sollte die Indikation zur CT aufgrund der **Strahlenexposition**

Strahlenexposition

streng gestellt werden. Sie ist insbesondere bei Verdacht auf intratemporale Komplikationen oder bei fehlender klinischer Besserung unter adäquater Therapie gegeben. Dies gilt v. a., wenn die Bildgebung für die weitere therapeutische und operative Planung erforderlich ist. In diesen Fällen überwiegt der diagnostische sowie für die operative Therapieplanung relevante Nutzen die Strahlenbelastung. Wenn möglich, sollten **Low-Dose-CT-Protokolle**

Low-Dose-CT-Protokolle

verwendet werden.

### Merke

Für intratemporale Befunde eignet sich primär eine CT-Bildgebung, für intrakranielle Komplikationen eine MRT-Untersuchung.

## Therapie und Management

Die Behandlung von Komplikationen der AOM richtet sich nach Art und Schwere der jeweiligen Komplikation sowie nach dem klinischen Zustand des Patienten. Ein rasches und zielgerichtetes Vorgehen ist von zentraler Bedeutung, um den Krankheitsverlauf zu kontrollieren und Folgeschäden zu verhindern.

Bei intratemporalen Komplikationen wie Mastoiditis, Labyrinthitis oder Fazialisparese steht zunächst die antibiotische Therapie im Vordergrund. **Empirische Breitbandantibiotika**

Empirische Breitbandantibiotika

werden i.v. verabreicht und anhand mikrobiologischer Befunde angepasst. Zusätzlich kann eine **Parazentese**

Parazentese,

ggf. mit **Paukendrainage**

Paukendrainage,

durchgeführt werden (bei Kindern ist zusätzlich eine Adenotomie zur Verbesserung der Tubenventilation zu erwägen); diese sollte(n) großzügig erfolgen, um vom Druck zu entlasten, Eiter zu drainieren und die Mittelohrbelüftung zu verbessern.

### Merke

Bei intratemporalen Komplikationen der AOM ist neben antibiotischer Therapie bei fehlender klinischer Besserung eine frühzeitige Parazentese/Paukendrainage indiziert.

Bei Labyrinthitis richtet sich das Management nach der klinischen Ausprägung. Die **seröse Labyrinthitis**

Seröse Labyrinthitis

wird primär konservativ mit antibiotischer Therapie i.v. behandelt und ist häufig reversibel. Die **eitrige Labyrinthitis**

Eitrige Labyrinthitis

hingegen ist mit einem hohen Risiko für irreversible sensorineurale Hörschädigung und persistierende vestibuläre Defizite verbunden. In diesen Fällen ist eine frühzeitige therapeutische Eskalation erforderlich, einschließlich engmaschiger klinischer und bildgebender Verlaufskontrolle. Bei progredientem Verlauf oder fehlendem Ansprechen auf die antibiotische Therapie ist eine **chirurgische Herdsanierung**

Chirurgische Herdsanierung

im Sinne einer Mastoidektomie zur Elimination des Infektionsfokus indiziert, um eine weitere Ausbreitung in Richtung intrakranieller Komplikationen zu verhindern. Bei eingetretener irreversibler sensorineuraler Hörschädigung sollte frühzeitig eine audiologische Abklärung und Einleitung rehabilitativer Maßnahmen erfolgen, einschließlich **Hörgeräteversorgung**

Hörgeräteversorgung

oder **Cochleaimplantation**

Cochleaimplantation

bei entsprechender Indikation. Eine operative Therapie bei Fazialisparese, etwa Mastoidektomie mit oder ohne **Fazialisnervdekompression**

**Abb. 3:**
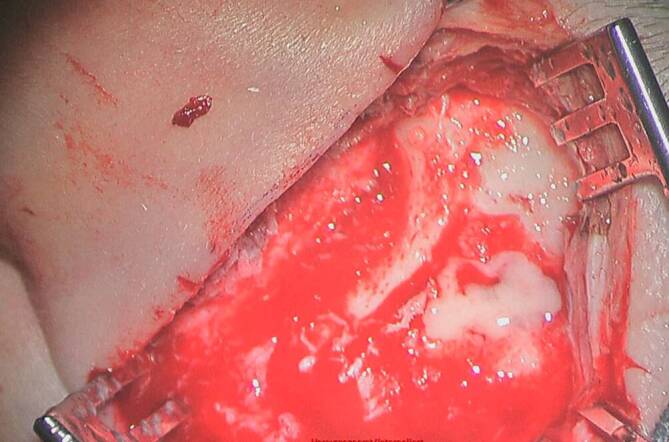
Intraoperatives Bild einer Mastoidektomie auf der linken Seite mit Eiteraustritt

Fazialisnervdekompression,

wird in der klinischen Praxis häufig erwogen, insbesondere bei kompletter oder progredienter Parese; in der Literatur wird der Eingriff jedoch kontrovers diskutiert. Während eine isolierte Mastoidzellverschattung in der Bildgebung häufig lediglich einer mukosalen Entzündung im Rahmen einer akuten Otitis media entspricht und keine Operationsindikation darstellt, besteht eine Indikation zur Mastoidektomie insbesondere bei fehlendem Ansprechen auf eine adäquate antibiotische Therapie oder beim Auftreten von subperiostalen bzw. extratemporalen Abszessen, die einer chirurgischen Drainage bedürfen. Ebenso ist die operative Therapie bei intrakraniellen Komplikationen indiziert. Bei schweren intratemporalen Komplikationen kann eine operative Sanierung des Mittelohrs und Mastoids ebenfalls erforderlich sein, insbesondere bei klinisch relevanter Fazialisparese (House-Brackmann Grad IV–VI oder bei progredientem Verlauf trotz antibiotischer Therapie) sowie bei eitriger Labyrinthitis im Rahmen einer komplizierten Otitis media oder Mastoiditis mit fehlendem klinischem Ansprechen auf konservative Therapie. Bei klinisch fortschreitender Mastoiditis unter konservativer Therapie ist eine operative Herdsanierung im Sinne einer Mastoidektomie (Abb. 3) ebenfalls indiziert [19, 28].

### Merke

Bei Abszessbildung oder progredientem Verlauf trotz adäquater Therapie ist eine operative Herdsanierung mit Mastoidektomie und chirurgischer Drainage erforderlich.

Intrakranielle Komplikationen erfordern häufig ein interdisziplinäres chirurgisches Vorgehen zwischen HNO-Heilkunde und **Neurochirurgie**

Neurochirurgie.

Bei Abszessen ist die chirurgische Drainage oft unverzichtbar, ergänzt durch eine hochdosierte i.v.-Antibiotikatherapie. Bei Sinusvenenthrombosen können zusätzlich **Antikoagulanzien**

Antikoagulanzien

indiziert sein, während Meningitiden eine sofortige antibiotische Behandlung und ggf. supportive Therapie auf der **Intensivstation**

Intensivstation

erfordern. Im klinischen Alltag kommen bei intrakraniellen Komplikationen typischerweise **Cephalosporine**

Cephalosporine

der dritten Generation, wie Ceftriaxon, zum Einsatz, da sie ein breites Erregerspektrum abdecken und liquorgängig sind [22, 28, 29, 30]. Bei Meningitis erfolgt die kalkulierte antibiotische Initialtherapie standardmäßig in Kombination mit Ampicillin [30]. Aufgrund des Risikos einer **raschen Labyrinthverknöcherung**

Rasche Labyrinthverknöcherung

sollte bei **meningitisassoziierter Hörminderung**

Meningitisassoziierte Hörminderung

frühzeitig eine Evaluation hinsichtlich einer Cochleaimplantation erfolgen [31].

Parallel zu gezielten Maßnahmen ist eine engmaschige klinische Kontrolle essenziell, um den Verlauf der Komplikation zu überwachen. Dabei sollten sowohl lokale Symptome (z. B. Ohrenschmerzen, retroaurikuläre Schwellung oder Schwellungen in Hals- und Nackenbereich bei Abszessen) als auch systemische Zeichen (Fieber, neurologische Auffälligkeiten) berücksichtigt werden.

Insgesamt zeigt sich, dass die erfolgreiche Therapie von AOM-Komplikationen ein schnelles Erkennen, angemessenes antibiotisches Management, ggf. chirurgisches Eingreifen und interdisziplinäre Koordination erfordert. Durch diese **strukturierte Vorgehensweise**

Strukturierte Vorgehensweise

lassen sich schwere Verläufe vermeiden und die Genesung der Patienten beschleunigen.

### Merke

Intrakranielle Komplikationen erfordern ein sofortiges interdisziplinäres chirurgisches Management.

## Prognose

Die Prognose komplizierter Verläufe der AOM hängt entscheidend vom frühen Erkennen und der raschen Einleitung einer adäquaten Therapie ab. Intratemporale Komplikationen zeigen i. d. R. eine gute Erholung ohne dauerhafte funktionelle Defizite. Dennoch können in einem Teil der Fälle Residuen wie **persistierende Hörschädigungen**

Persistierende Hörschädigungen

auftreten. Intrakranielle Komplikationen sind mit deutlich höherer Morbidität und auch erhöhter Mortalität verbunden; neurologische oder **funktionelle Restdefizite**

Funktionelle Restdefizite

sind hier häufiger. Adäquate diagnostische und therapeutische Maßnahmen reduzieren schwere Verläufe deutlich. Eine **engmaschige Überwachung**

Engmaschige Überwachung

sowie eine strukturierte interdisziplinäre Versorgung tragen maßgeblich zu günstigen funktionellen Outcomes bei [6, 28].

## Fazit für die Praxis

- Komplikationen der akuten Otitis media (AOM) sind selten, erfordern jedoch aufgrund potenziell schwerer Verläufe eine hohe klinische Aufmerksamkeit.
- Die Diagnose beruht auf klinischen Warnzeichen in Kombination mit Laborparametern und gezielter Bildgebung für eine sichere Differenzierung der Komplikationen.
- Intratemporale Komplikationen erfordern ein stufenweises Vorgehen: Initial erfolgt eine antibiotische Therapie. Bei persistierendem Paukenerguss und fehlender klinischer Besserung ist eine frühzeitige Parazentese, ggf. mit Paukendrainage (bei Kindern ggf. kombiniert mit einer Adenotomie), indiziert. Bei Abszessbildung oder fehlendem Ansprechen auf die Therapie ist eine Mastoidektomie mit suffizienter Drainage des Infektionsherds erforderlich.
- Intrakranielle Komplikationen erfordern häufig eine interdisziplinäre chirurgische Behandlung zwischen HNO-Heilkunde und Neurochirurgie.
- Engmaschige Verlaufskontrolle und interdisziplinäre Abstimmung sind entscheidend, um schwere Verläufe frühzeitig zu erkennen und die Prognose zu verbessern.

## Data Availability

Alle dieser Arbeit zugrunde liegenden Daten sind in diesem Artikel enthalten.

## CME-Fragebogen

Ein 3‑jähriges Kind mit rezidivierender Otitis media präsentiert sich mit hohem Fieber und retroaurikulärer Schwellung. Welche Komplikation ist am wahrscheinlichsten?

Labyrinthitis

Mastoiditis

Fazialisparese

Sinusvenenthrombose

Meningitis

Welche Aussage zu intrakranialen Komplikationen der akuten Otitis media (AOM) trifft zu?

Sie entstehen durch direkte Ausbreitung über das submuköse Fettgewebe.

Subdurale oder epidurale Abszesse sind häufige intratemporale Komplikationen.

Sinusvenenthrombosen können als Folge einer venösen Ausbreitung auftreten.

Intrakranielle Komplikationen sind meist asymptomatisch.

Labyrinthitis zählt zu den intrakraniellen Komplikationen.

Bei Verdacht auf eine intrakraniale Komplikation der akuten Otitis media (AOM) sollte welches bildgebende Verfahren primär durchgeführt werden?

Magnetresonanztomographie (MRT) des Schädels mit Kontrastmittel

Röntgenaufnahme des Schädels

Computertomographie (CT) des Felsenbeins

Ultraschall der Halsweichteile

Fluordesoxyglukose-Positronenemissionstomographie-Computertomographie (FDG-PET-CT)

Welche Warnzeichen deuten auf eine komplizierte Otitis media hin?

Leichte Ohrenschmerzen und gelegentliches Fieber

Retroaurikuläre Schwellung und neurologische Auffälligkeiten

Rhinitis und leichtes Ohrgeräusch

Periodische Tinnitusattacken

Kurzzeitige Schwindelanfälle ohne andere Symptome

Bei einem Erwachsenen mit Diabetes mellitus entwickelt sich eine akute Otitis media (AOM) mit plötzlicher Fazialisparese. Welche Maßnahme ist vorrangig?

Langzeit-Antibiotikatherapie p.o.

Engmaschige Kontrolle, konservative Behandlung

Intravenöse antibiotische Therapie, Bildgebung

Hörgeräteversorgung

Schmerztherapie ausreichend

Welche Aussage zur antibiotischen Therapie intratemporaler Komplikationen trifft zu?

Orale Antibiotika sind meist ausreichend.

Breites Spektrum und i.v.-Gabe werden empfohlen.

Antibiotika sind unterstützend, die chirurgische Therapie ist obligat.

Topische Ohrentropfen reichen in den meisten Fällen aus.

Antibiotika sind bei intrakraniellen Komplikationen nicht indiziert.

Ein 5‑jähriges Mädchen wird wegen einer akuten Otitis media seit 3 Tagen mit einem Antibiotikum behandelt. Die Eltern berichten über eine leichte Besserung der Ohrenschmerzen und rückläufiges Fieber. Bei der Untersuchung zeigt sich ein gerötetes Trommelfell ohne retroaurikuläre Schwellung. Die Ohrmuschel steht nicht ab. Das Kind ist wach, altersentsprechend orientiert und zeigt keine neurologischen Auffälligkeiten. Laborchemisch finden sich ein Wert für C‑reaktives Protein (CRP) von 45 mg/l und eine Leukozytose von 14.000/µl. Welche diagnostische Maßnahme ist aktuell am ehesten angezeigt?

Kontrastmittelgestützte Computertomographie (CT) des Felsenbeins zur frühzeitigen Erkennung einer Mastoiditis

Kontrastmittelgestützte Magnetresonanztomographie (MRT) des Schädels mit MR-Venographie

Sofortige operative Mastoidektomie

Klinische Verlaufskontrolle unter Fortführung der Therapie

Lumbalpunktion zum Ausschluss einer Meningitis

Welche Veränderungen lassen sich bei einer Mastoiditis in der Computertomographie (CT) des Felsenbeins erkennen?

Ossifikation der Cochlea

Luftgefüllte Mastoidzellen ohne Flüssigkeit

Knochenresorption im Mastoidbereich

Erweiterter Fazialiskanal

Subduraler Abszess

Welche Patientengruppe hat ein erhöhtes Risiko für komplizierte Verläufe?

Gesunde Erwachsene

Kinder unter 5 Jahren mit rezidivierender Otitis media

Jugendliche ohne Vorerkrankungen

Erwachsene mit saisonaler Rhinitis

Leistungssportler

Welche Maßnahme ist zentral bei der Behandlung intrakranieller Komplikationen?

Orale Antibiotikatherapie

Interdisziplinäre chirurgische Behandlung

Schmerztherapie und Beobachtung

Topische Ohrentropfen

Hörgeräteversorgung
